# Uncovering mercury accumulation and the potential for bacterial bioremediation in response to contamination in the Singalila National Park

**DOI:** 10.1038/s41598-024-81927-5

**Published:** 2025-01-29

**Authors:** Sukanya Acharyya, Soumya Majumder, Sudeshna Nandi, Arindam Ghosh, Sumedha Saha, Malay Bhattacharya

**Affiliations:** Molecular Biology and Tissue Culture Laboratory, Department of Tea Science, University of North Bengal, Siliguri, West Bengal India

**Keywords:** Mercury, Himalayas, Soil pollution, MRB, Singalila National Park, Mountain trapping, Microbiology, Environmental sciences

## Abstract

Several recent investigations into montane regions have reported on excess mercury accumulation in high-altitude forest ecosystems. This study explored the Singalila National Park, located on the Singalila ridge of the Eastern Himalayas, revealing substantial mercury contamination. Particular focus was on Sandakphu (3636 m), the highest peak in West Bengal, India. It harboured 6.77 ± 0.01 mg/kg of total mercury in its topsoil. Further evidence was provided by accumulation in leaves (0.040 ± 0.01 mg/kg), and roots (0.150 ± 0.008 mg/kg) of local vegetation, litterfall (0.234 ± 0.019 mg/kg), mosses (0.367 ± 0.043 mg/kg), surface water from local lakes and waterbodies (0.010 ± 0.005 mg/l), fresh snow (0.014 ± 0.004 mg/l), and sleet (0.019 ± 0.009 mg/l). Samples from other points of varying elevation in the park also demonstrated contamination. The soil displayed a range of 0.068–5.28 mg/kg, while the mean concentration in leaves was 0.153 ± 0.105 mg/kg, roots was 0.106 ± 0.054 mg/kg, and leaf litter was 0.240 ± 0.112 mg/kg. Additionally, the microbial consortia isolated from the contaminated soil displayed a high tolerance to mercuric chloride, presumably gained through repeated and consistent exposure. Four high tolerance bacterial strains, MTS2C, MTS3A, MTS4B and MTS6A, were further characterized for potential use in bioremediation strategies. Their mercury removal capacities were determined to be 82.35%, 75.21%, 61.95%, and 37.47%, respectively. Overall, the findings presented provide evidence for a highly contaminated environment in the Singalila National Park, that poses significant ecological risk to the flora, fauna and local inhabitants of this biodiversity hotspot. This research also highlights the need for further exploration and monitoring of the Eastern Himalayas for its role as a sink for atmospheric mercury.

**Supplementary Information:**

The online version contains supplementary material available at 10.1038/s41598-024-81927-5.

## Introduction

Mercury is a highly toxic heavy metal pollutant, the presence of which is rising in the environment day by day^1^. It is ranked third on the priority list of hazardous chemicals^2^ due to the detrimental effects it can have on the health and survival of all living organisms. The complex biogeochemical cycling of this semi-volatile pollutant and the several factors governing its excess presence in the environment have been studied extensively. This has revealed unique modes of transport that facilitate the transfer of anthropogenic mercury from its original source to remote locations that harbour vulnerable ecosystems. Researchers have noted the presence of copious amounts of mercury, even at the poles^3,4^, generating concern over the distribution of, and, exposure to the pollutant. In this context, the accumulation of mercury in high-altitude environments has gained prominence. In recent years, several studies have reported the presence of mercury in montane regions^5–7^. The Tibetan Plateau, also known as the third pole of the world, has emerged as a potential sink for this heavy metal. A few studies have suggested contamination in parts of the Himalayas^7,8^. The sharp altitudinal gradients, wind trajectory, climactic variations, etc., characteristic of mountainous regions, offer a suitable mix of factors that enable the transfer and deposition of mercury at these locations. Montane regions demonstrate a phenomenon known as “mountain trapping” or “cold trapping”. This causes volatile or semi-volatile pollutants like mercury from warmer low-lying areas to be carried via the air into the mountains. Here, they encounter a colder climate and precipitate in atmospheric depletion events as rain or snow. The result is an accumulation of the pollutant in the mountain ecosystem, with repeated deposition over time.

This study aimed to explore one such area for the presence of mercury pollution, i.e., Sandakphu (3636 m), the highest peak of West Bengal, India. Sandakphu is located on the Singalila ridge, that falls within the Singalila National Park, on the north-west of Darjeeling. The ridge forms a natural boundary between India and Nepal and is a part of the Eastern Himalayas. This region is adjacent to one of the most polluted regions in the world (Indo-Gangetic plains) and some of the largest contributors to the global atmospheric mercury load i.e., East, South–East and South Asia^1^. The park has no prior history of human settlement and thus may be considered untouched by the hazardous byproducts of anthropogenic activities. Only very small settlements have formed over the years along a popular trekking route to Sandakphu that runs through the park. The nearest city is Maney Bhanjang (1928 m), about 32 kms from Sandakphu by road.

Along with the peak, several locations in the Singalila forest have been included in this study. Together they provide a steep altitudinal gradient (2400–3650 m) and climate variation (temperate to sub-alpine)^9^, conducive to the study of long-range transport of mercury. The complex topography of the park constitutes an intercalation of ridges and spurs that, responsible for the generation of many microclimates. Temperatures in the temperate zone of the park range from 7 to 17 °C in summer and 1 °C to 10 °C in winter, while the sub-alpine zone has a mean summer temperature under 7 °C and winter temperature below 1 °C^10^. Humidity is consistently high throughout the year at 83–96% and regular snowfall occurs at and above 3300 m. Frost covers almost 80% of the park from December to March. The area also receives a mean annual rainfall of 330 cm^11^. Moisture-laden southwest monsoon winds from the Bay of Bengal are carried straight into the region. The Singalila ridge forms a significant barrier to its flow, resulting in heavy rainfall, constant cloud cover and a humid climate on the eastern face of the ridge. This may be enabling the long-range transport, deposition, and cold-trapping of mercury in the area, making it a near perfect model for the exploration of this phenomenon.

## Results and discussion

### Mercury in soil

**Fig. 1:**
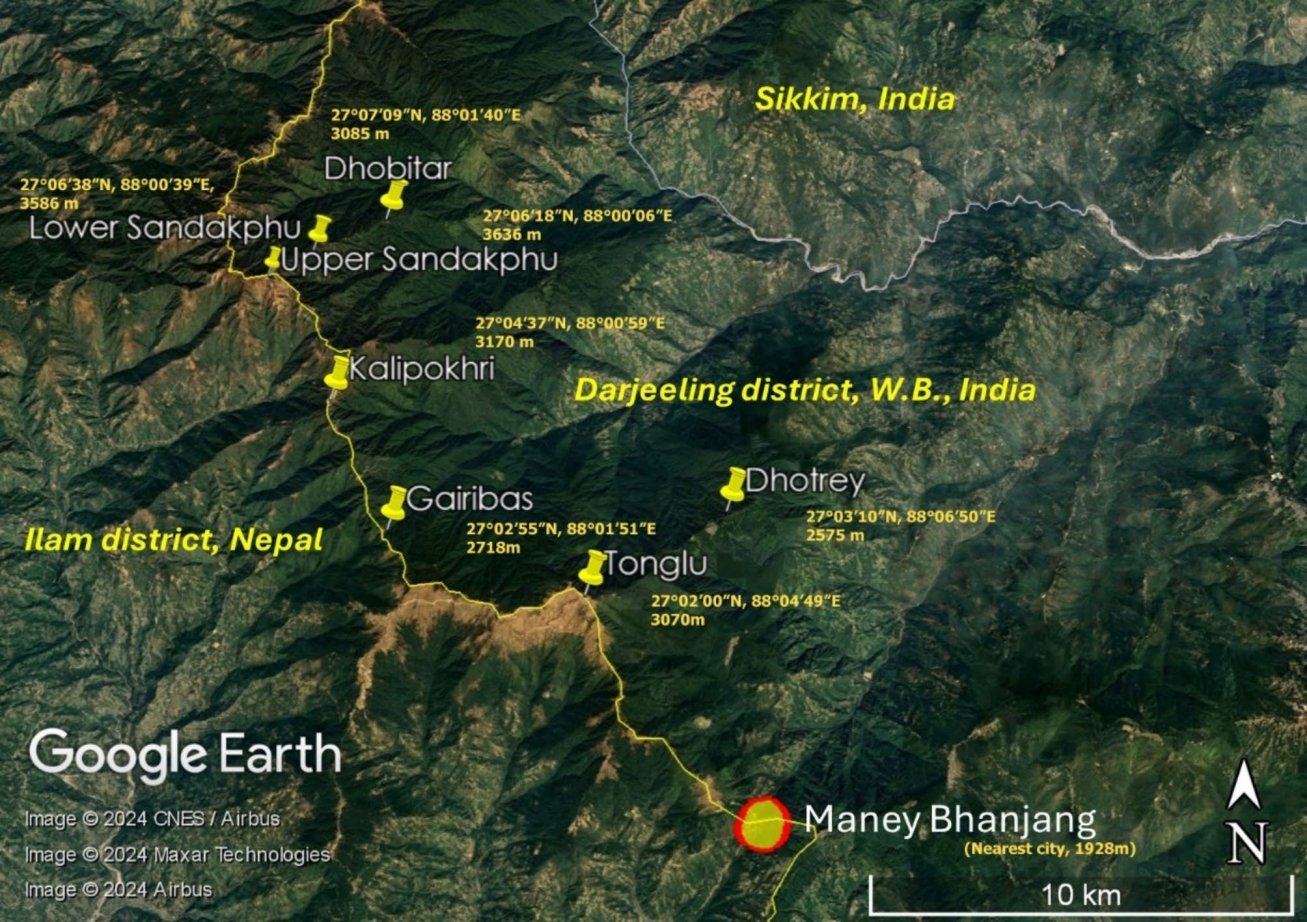
Map of the collection sites within the Singalila National Park.

**Fig. 2:**
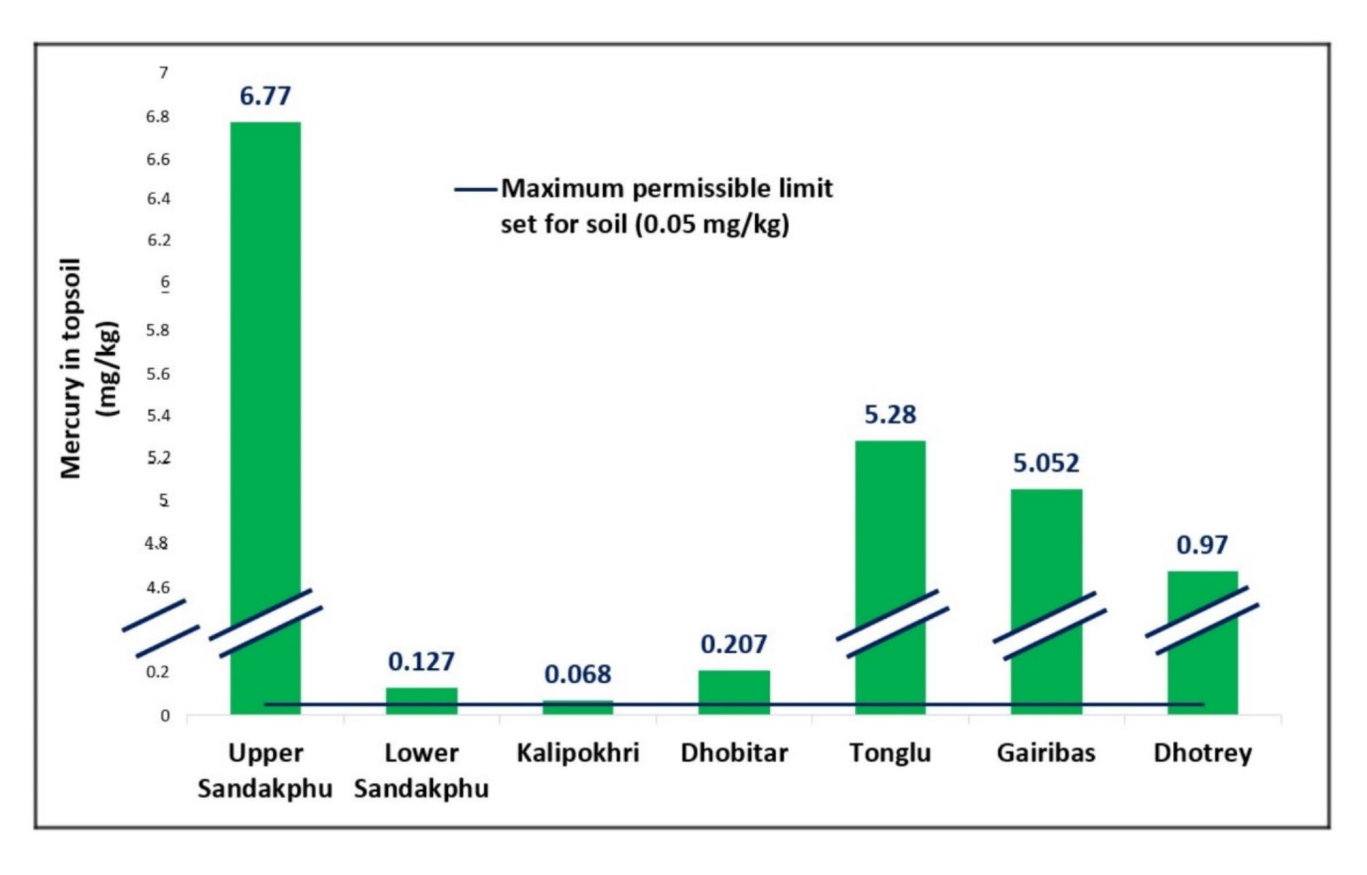
Graph representing the respective concentrations of total mercury detected in the topsoil from each collection site, in comparison to the maximum permissible limit jointly set by WHO and BIS (Bureau of Indian Standards), i.e., 0.05 mg/kg. The Y-axis represents the amount of mercury in mg/kg, and includes a break to highlight the differences in detected mercury content.

High level of mercury contamination was detected in the soil collected from the study area. Seven different sites with varying elevations were investigated (Fig. 1). The results showed a rampant distribution of mercury in the range of 0.068–6.770 mg/kg (Fig. 2). All the results obtained were many folds higher than 0.05 mg/kg^12^, the maximum permissible limit of mercury in soil jointly set by WHO and BIS (Bureau of Indian Standards), and provided indisputable evidence of contamination. Notably, the maximum mercury was found at the point of highest elevation, Sandakphu (6.770 ± 0.013 mg/kg). However, upon decent from the peak towards the south east, the lower Sandakphu soil collected, displayed a much lower mercury content (0.127 ± 0.011 mg/kg). This may be due to the collection site being densely forested, and located on an adjoining east-west oriented spur. Further along the same spur was Dhobitar, which also exhibited lesser mercury accumulation (0.207 ± 0.011 mg/kg), in comparison to the other collection sites. This area was covered in a dense bamboo forest. In the case of mercury cold-trapping, mercury is transported into the mountains via wind currents. The impact of these wind systems with regard to temperature, rainfall, and overall trajectory can vary greatly, even within very small distances^13^. It is further influenced by the unique configuration of the neighbouring mountain ranges. The lower Sandakphu sampling location, and Dhobitar, were both located on a descending route from the main Singalila ridge. As such, they may be protected from higher atmospheric mercury deposition due to some influence of the neighbouring ranges on the dynamics of the wind currents. The dense forest cover over these locations may also be responsible for restricting the amount of mercury reaching the soil. Furthermore, the lower Sandakphu site was particularly steep, with a loose top layer of soil. This may have caused loss of deposited mercury due to simple erosion.

The second highest concentration of mercury was found at another high point on the ridge, i.e., Tonglu (5.280 ± 0.011 mg/kg). The sediment sample collected separately from Tonglu lake, had 0.125 ± 0.012 mg/kg of mercury, which although high, was much less than the terrestrial topsoil sample. This may be due to limited mobility of mercury in the environment. Gairibas closely followed Tonglu in mercury content (5.052 ± 0.012 mg/kg). Gairibas hosts a very humid climate from being positioned in a valley, surrounded by dense forests. It receives heavy rainfall that can contribute significantly to the deposition of mercury, and, remains shrouded in mist/fog. This restricts solar radiation that can promote reemission through surface photoredox reactions and revolatilization. It is estimated that about 5–60% of mercury can be recycled into the atmosphere after deposition^14^. Thus, lower incidence of solar radiation facilitates retention of deposited mercury in the soil. The lowest point explored in this study, i.e., Dhotrey also displayed considerable contamination (0.970 ± 0.016 mg/kg), but not the lowest. Interestingly, the minimum mercury content was detected at another one of the higher elevation points, i.e., Kalipokhri (0.068 ± 0.011 mg/kg). The global background range for mercury in soil falls within 0.003–0.060 mg/kg^15^. Only Kalipokhri displayed mercury that was close to this range. Additionally, the sediment sample collected from lake Kalipokhri also displayed low mercury (0.060 ± 0.011 mg/kg). This may be due to prompt reemission of deposited mercury after deposition.

The characterization of the soil samples (Table 1) revealed them to be acidic in nature, typical in these regions. Acidic soil may encourage formation of mercury complexes with soil organic matter, leading to enhanced retention. It also enhances mobility, as well as, potential for transformation into more toxic, organic forms^16^. A weak positive correlation (0.20) was found between the pH and the mercury content in the studied soil and sediment samples. The samples also displayed high organic carbon content. Higher organic carbon has been linked to the promotion of microbes responsible for the conversion and cycling of the highly toxic methylmercury^17,18^. Furthermore, the organic matter content in all the samples was high (> 5%), except in Kalipokhri. Mercury is primarily deposited onto soil in the inorganic form and forms very strong associations with humic substances in the case of acidic soil. These relationships discourage remission and promote retention. Mercury humic complexes have high stability, especially complexes with humic acids (associations with O-ligands) and fulvic acids (associations with carboxyl groups)^16^. The impact of such associations can be observed when comparing differences in mercury content at Tonglu and Kalipokhri. Although both sites share a similar physio-geography, Tonglu displayed much higher mercury content than Kalipokhri. This may be due to the much higher organic matter content of Tonglu (21.19%) than that of Kalipokhri (3.85%). The low organic matter at Kalipokhri may be unable to capture mercury efficiently. A moderate positive correlation (0.40) was observed between the organic matter and mercury content of the soil and sediment samples. Soils with low organic matter have also been shown to harbour more reactive mercury that is prone to methylation^19^.

**Table 1:** Physico–chemical characters of soil and sediment samples.

Location	pH	S (ppm)	OC (%)	OM (%)	*N* (%)	K (ppm)
Upper Sandakphu topsoil	6.39 ± 0.3	22.32 ± 0.22	5.43 ± 0.11	9.34 ± 0.07	0.46 ± 0.5	285 ± 12
Lower Sandakphu topsoil	4.68 ± 0.2	19.98 ± 0.30	6.23 ± 0.08	10.72 ± 0.06	0.53 ± 0.4	140 ± 26
Kalipokhri topsoil	5.37 ± 0.3	19.19 ± 0.15	2.24 ± 0.09	3.85 ± 0.06	0.19 ± 0.4	175 ± 18
Kalipokhri lake sediment	6.04 ± 0.2	17.83 ± 0.08	0.72 ± 0.06	1.24 ± 0.18	0.06 ± 0.2	105 ± 31
Dhobitar topsoil	5.23 ± 0.1	24.61 ± 0.34	10.15 ± 0.51	17.45 ± 0.12	0.87 ± 0.6	365 ± 07
Tonglu topsoil	4.52 ± 0.2	24.68 ± 0.23	12.32 ± 0.06	21.19 ± 0.16	1.05 ± 0.3	445 ± 24
Tonglu lake sediment	5.35 ± 0.2	20.53 ± 0.08	1.99 ± 0.12	3.42 ± 0.23	0.17 ± 0.7	220 ± 27
Gairibas topsoil	5.21 ± 0.1	20.57 ± 0.39	5.90 ± 0.11	10.15 ± 0.09	0.50 ± 0.4	300 ± 36
Dhotrey topsoil	4.58 ± 0.2	21.90 ± 0.50	7.06 ± 0.19	12.14 ± 0.09	0.60 ± 0.7	165 ± 17

*S* sulphur, *OC* organic carbon, *OM* organic matter, *N* nitrogen, *K* potassium.

The sulphur content did not show a great degree of variability among the different soil samples. However, a moderate positive correlation (0.42) was displayed between the sulphur and mercury content in the soils. Deposited mercury is known to readily form very strong associations with reduced sulphur due to high affinity^20^. In organic matter, mercury relies on binding to functional groups like the sulphur containing thiols as it cannot bind to C directly^20^. The difference in interactions is exemplified by the two sediment samples collected from the lakes at Tonglu and Kalipokhri. These were both high in organic matter but very low in their sulphur content. Corresponding to this, their detected mercury content was also much lower compared to the other samples (Tonglu lake sediment, 0.125 ± 0.012 mg/kg; Kalipokhri lake sediment, 0.060 ± 0.011 mg/kg). Furthermore, a strong positive correlation (0.61) was displayed between the potassium and mercury content in the studied samples. Thus, a complex interplay of several factors governs the retention/reemission balance of mercury in an ecosystem. Much more study is needed to conclusively state the reasons for variations in mercury content at each location.

### Mercury in vegetation

**Fig. 3:**
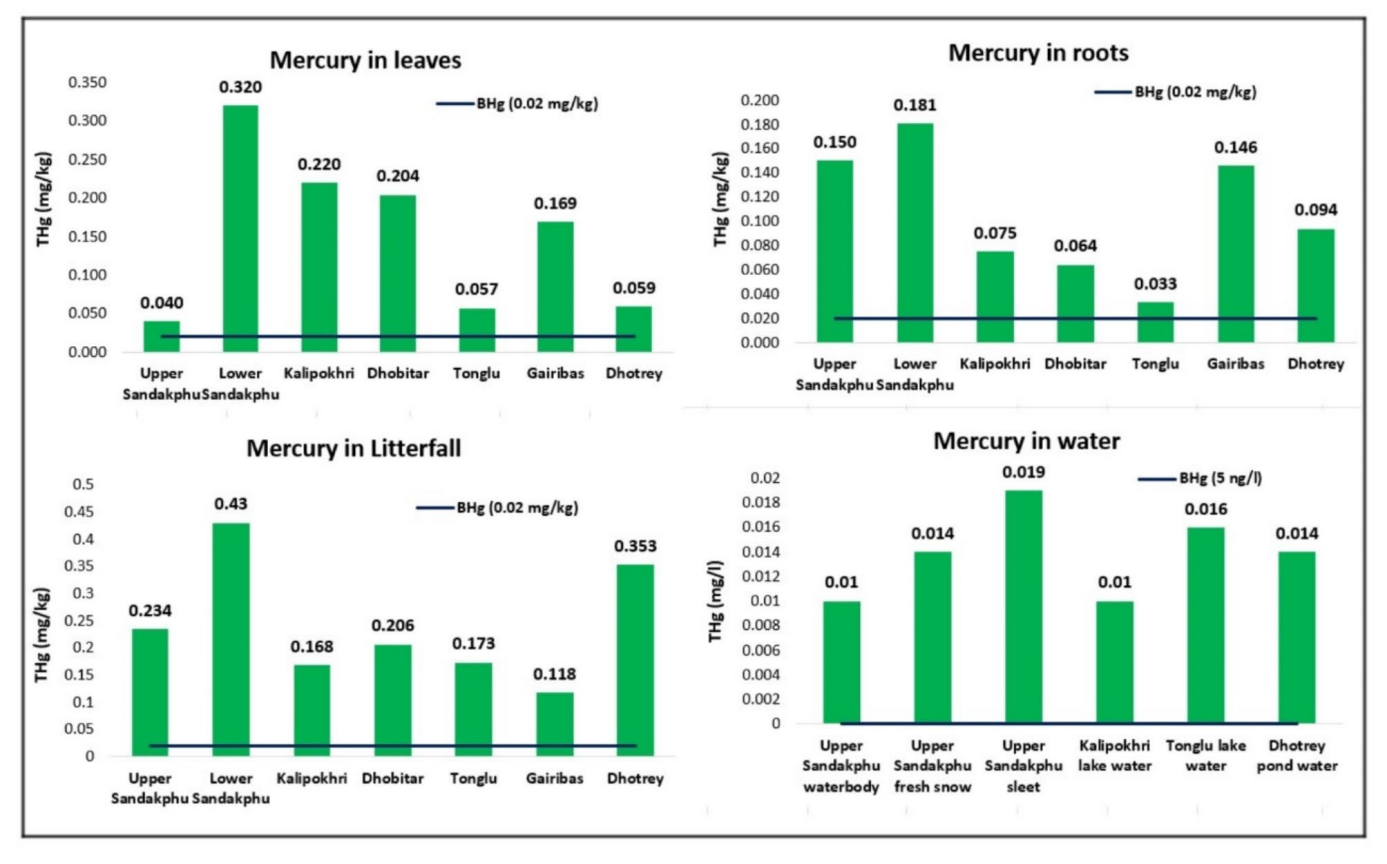
Graphs representing the concentration of total mercury (THg) detected in different vegetation and other types of samples collected at each studied location. The line data series ‘BHg’ represents the threshold concentrations of mercury permissible in food (in case of leaf, litterfall, root, and moss samples), water (for freshwater from lakes and waterbodies), used for better visualization of degree of contamination. The threshold values used were 0.02 mg/kg for all vegetation samples (EFSA), and 5 ng/l for water samples, respectively.

Proof of mercury contamination was also present in the quantification data obtained for vegetation samples from the region (Fig. 3). It is important to note that the samples were only cleaned superficially, and not methodically to remove traces of mercury on their surface. Thus, any mercury detected represents the collective load of mercury both in and on the samples. Collected leaves displayed a mean concentration of 0.153 ± 0.105 mg/kg, while the roots displayed 0.106 ± 0.054 mg/kg. The lower concentration of mercury detected in the leaves in comparison to the extremely high mercury present in the soil of upper Sandakphu, may have been a result of washing away of mercury deposited on the surface of the leaves by the snowfall before collection. The mean concentration displayed by the leaf litter samples was at 0.240 ± 0.112 mg/kg. All the samples individually exhibited exceeding concentrations of mercury, far beyond the permissible limit of 0.020 mg/kg^21^ (Fig. 3). Separate collections of broad and narrow leaves were made from the upper Sandakphu collection site for comparison in accumulation capacity. However, both displayed similar values, with the broad leaves having 0.040 ± 0.01 mg/kg, and narrow leaves having 0.048 ± 0.012 mg/kg of mercury respectively. In measuring mercury contamination of forest ecosystems, litterfall is considered an important source that significantly contributes to the soil pool. The decomposition process does not affect the isotopic composition of the sequestered mercury in the fallen leaves^22^, and gets added to the soil in its original form. The litterfall collected from upper Sandakphu demonstrated a high concentration of mercury at 0.234 ± 0.019 mg/kg. This is a good indication of the continued assimilation of mercury in the leaves over time. However, some studies have suggested that litterfall does not provide the best indication of mercury deposition in an ecosystem as it does not account for mercury assimilated by the woody tissue of vascular plants. Mosses have been shown to be a far better bioindicator of atmospheric mercury, subject to species and the microhabitat^23^. This is due to the fact that accumulation in mosses occurs largely through direct assimilation from the air^24^. The moss sample collected from Sandakphu displayed a much higher accumulation at 0.367 ± 0.043 mg/kg. Mercury in moss and lichen samples from Kodaikanal, India, marked as one of the 35 heavily contaminated sites in the country in need of urgent remediation, have reported lower concentrations of mercury at 0.2 mg/kg than the sample from Sandakphu. Thus, it may be considered evidence for the presence of gaseous mercury (Hg^0^) in the air mass over the region.

Interestingly, a considerable amount of mercury was detected in the leaves from Kalipokhri (0.220 ± 0.031 mg/kg), despite the Kalipokhri topsoil having displayed the lowest concentration of the metal. Low mercury in the soil would not result in such high accumulation in leaves if it were solely reliant on uptake via the roots. As a matter of fact, mercury detected in the roots themselves was much lower (0.075 ± 0.013 mg/kg). Therefore, the high accumulation may be a result of the plants conducting direct uptake of Hg^0^ via the stomata and cuticle^25^. Uptake in this way is further enhanced when intercepting cloud or fog, which have reportedly displayed higher levels of mercury than precipitation^26^. Higher than expected accumulation was also observed in the vegetation samples from lower Sandakphu and Dhobitar, both of which had demonstrated lower mercury in their soil. The foliar samples from both sites displayed higher mercury content (lower Sandakphu, 0.320 ± 0.031 mg/kg; Dhobitar, 0.204 ± 0.010 mg/kg), than their roots (lower Sandakphu, 0.181 ± 0.017 mg/kg; Dhobitar, 0.064 ± 0.016 mg/kg). The leaf litter samples had also accumulated high levels of mercury at these locations (lower Sandakphu, 0.430 ± 0.043 mg/kg; Dhobitar, 0.206 ± 0.014 mg/kg). As leaf litter does not account for re-emission dynamics from the soil pool, this may be further proof of atmospheric mercury deposition.

### Mercury in precipitation

Mercury was detected in the freshly precipitating snow (0.014 ± 0.004 mg/l) and the sample of sleet collected (0.019 ± 0.009 mg/l) at upper Sandakphu (Fig. 3). The presence of mercury in either snow or sleet, where there should be none, may be considered definitive evidence for atmospheric transport and deposition of mercury through precipitation in the region. In fact, the high concentration of mercury detected in the snow, may have influenced the high amount of mercury detected in the soil from upper Sandakphu, as this sample was collected after a fresh bout of overnight snowfall. Mercury was markedly absent in the older snow sample collected from the lower Sandakphu collection site. This sample was of undisturbed snow that had fallen a couple of days before the date of collection. The lower mercury detected may be due to it having re-emitted back into the atmosphere via photo-redox reactions, on account of it being an older deposit. As discussed earlier, a significant amount of mercury deposited can be promptly emitted back into the atmosphere under the influence of various processes and solar radiation. This flux has been reported to be greatly enhanced in snow^27^. Mercury concentrations in surface snow has been shown to be very dynamic and a negative relationship has been proposed with atmospheric mercury connected to diurnal solar radiation cycles. Approximately 90% reduction in mercury has been observed over a period of as little as 4–6 h after deposition. Studies on snowpacks from the Arctic ecosystem have demonstrated the rapid reduction and revolatilization of deposited mercury^28^. Thus, older snow deposits have little retention capacity for mercury after deposition.

### Mercury in waterbodies

All four water samples collected for mercury assessment displayed very similar values (Fig. 3). The water from Kalipokhri lake and the upper Sandakphu man made waterbody, displayed 0.010 ± 0.005 mg/l and 0.010 ± 0.008 mg/l of mercury, whereas, the Tonglu lake and water from Dhotrey had slightly higher levels at 0.016 ± 0.007 mg/l and 0.014 ± 0.008 mg/l, respectively (Fig. 3). Mercury is known to reach freshwater ecosystems predominantly as inorganic mercury, through wet/dry deposition onto lake surfaces or as runoff from the surrounding environment. Thereafter, sinking and adsorption to components in the soil or remineralization, is crucial to determining mercury available at the surface. This is especially true under low pH conditions^29^. A probable indication of removal by sedimentation may be seen in samples from the two lakes, i.e., Tonglu and Kalipokhri, which displayed higher mercury content in their lake sediments, compared to surface water. However, direct comparisons between mercury content in water and sediment cannot be made as there may be a host of mechanisms at play that affect the distribution. Mercury present in surface water engages in surface flux dynamics and an array of photo or biologically induced transformations that determine its overall distribution. Vertical column profiles measured have demonstrated a nutrient-like distribution of mercury, i.e., an increasing trend in concentration with increase in depth^30^. Thus, further study is needed to conclusively state the underlying reasons behind the apparent similar distribution of mercury in waterbodies located at varying altitudes. Regardless, it is important to note that only about 5 ng/l of mercury is thought to be naturally present in such water bodies^31^, many folds lower than what was seen at these locations. The lakes at Tonglu and Kalipokhri, or the waterbody at Dhotrey, serve as important sources of drinking water to the local wildlife species, many of which are endangered and protected within the park. Thus, the contamination of these may directly affect their health and wellbeing.

### Mercury tolerance in microbial consortia

Tolerance to a pollutant displayed by the resident microbiota of a location can be considered a significant indication of pollution, as tolerance is generally acquired through repeated exposure to the pollutant. In case of mercury, its presence in an environment at a concentration of 0.036 mg/kg is proposed to be enough for the alteration of the microbiome of that location^32^. With much higher concentrations already found to be present in the soil of the studied locations, the microbial consortia isolated also predictably displayed prolific growth at the highest concentration of added mercury tested, i.e., 0.05 mg/ml. This demonstrated the tangible impact of mercury exposure on resident microbiota of the study area. It is important to note that although some microbes evolve to become tolerant under the continued stress of such heavy metals, most do not survive. These circumstances adversely affect nutrient cycling in the soil by killing beneficial microbes and promote the spread of potentially harmful mercury methylating bacterial strains. Hence, mercury pollution critically influences microbial diversity, and by extension, the delicate balance of an ecosystem.

### Ecological risk assessment

The total mercury quantification data obtained for the soil samples were used to assess the extent of ecological risk posed to the region (Table 2). The enrichment factor (EF) determined was in the extreme enrichment range at upper Sandakphu, Tonglu and Gairibas, very high at Dhotrey, moderately high at Dhobitar, moderate at lower Sandakphu, and minor at Kalipokhri. The geoaccumulation index (I_geo_) and contamination factor (C_f_), further consolidated the pollution status of upper Sandakphu, Tonglu and Gairibas, which displayed moderate pollution and very high contamination. Finally, the overall ecological risk was calculated which illustrated the potential for harm to the living organisms of this region. Upper Sandakphu, Tonglu and Gairibas posed the maximum risk, while Dhotrey displayed moderate to high risk. The results obtained were highly concerning as the locations studied all fell within the Singalila National Park, one of the sixteen protected areas in the Khangchendzonga landscape, Eastern Himalayas. The region is home to many important species of plants, animals and indigenous communities that depend on the resources of these forests for sustenance and survival. The animal inhabitants of the region include the red panda, Himalayan black bear, Himalayan newt, clouded leopard, wild boar, barking deer, yellow-throated marten, pangolin, etc. It is also home to over 120 species of rare and exotic birds. Thus, the accumulation of mercury in these parts poses a direct threat to their health and wellbeing.

**Table 2:** Ecological risk assessment.

Location	EF	Enrichment	I_geo_	Category	C_f_	Contamination	E_*r*_	Risk
Upper Sandakphu	180.88	Extreme	0.85	Moderately polluted	16.92	Very high	677	Very high
Lower Sandakphu	3.39	Moderate	−4.88	Unpolluted	0.32	Low	12.7	Low
Kalipokhri	1.82	Minor	−5.78	Unpolluted	0.17	Low	6.8	Low
Dhobitar	5.53	Moderately high	−4.18	Unpolluted	0.52	Low	20.7	Low
Tonglu	141.07	Extreme	0.49	Moderately polluted	13.20	Very high	528	Very high
Gairibas	134.98	Extreme	0.43	Moderately polluted	12.63	Very high	505.2	Very high
Dhotrey	25.92	Very high	−1.95	Unpolluted	2.42	Moderate	97	Moderate to high

*EF* enrichment factor, *I*_*geo*_ geoaccumulation index, *C*_*f*_ contamination factor, *E*_*r*_ ecological risk factor.

### Isolation of mercury-remediating bacterial strains

As already evidenced from the assessment of the microbial consortia, tolerance to mercury was rampant among the microbes. Isolation of particular high tolerance strains of bacteria was carried out, from these consortia, to explore and exploit their potential for bioremediation. The determination of the minimum inhibitory concentration (MIC) helped select four strains, named, MTS2C, MTS3A, MTS4B, and MTS6A, that displayed particularly high tolerance to mercury. Their MICs were determined to be at 0.3 mg/ml, 0.2 mg/ml, 0.09 mg/ml and 0.1 mg/ml, respectively. The mercury removal capacities of the strains were at 82.35%, 75.21%, 61.95%, and 37.47%, respectively (Table 3). MTS2C and MTS3A displayed the best potential for removal of mercury from their surrounding environment. With further study, these strains may prove to be very useful in the design of remediation tactics against mercury contaminated terrestrial environments.

**Table 3:** Characterization of selected mercury tolerant strains.

Test	MTS2C	MTS3A	MTS4B	MTS6A
Pigmentation	Cream	Light orange	Cream	White
Colony morphology	Round, convex, S-type	Round, shiny, convex	Round, shiny, convex	Shiny, convex, watery
Identity	*Staphylococcus sciuri*	*Staphylococcus arlettae*	*Staphylococcus cohnii*	*Lactobacillus salivarius*
Homology	99.89%	99.92%	99.77%	99.46%
Accession number	PP415850	PP436698	PP416278	PP815117
MIC (mg/ml)	0.3	0.2	0.09	0.1
Mercury removal	82.35%	75.21%	61.95%	37.47%
Gram stain	+	+	+	+
Morphology	Cocci	Cocci	Paired cocci	Cocci
Methyl red test	+	+	+	−
Citrate	−	−	−	−
Coagulase	−	−	−	−
Gelatin hydrolysis	+	+	−	−
Nitrate reduction	+	−	−	−
Urease test	−	−	+	−
Voges Proskauer	−	−	−	−
Motility test (SIM)	−	−	−	−
Starch hydrolysis	−	−	+	+
Catalase	+	+	+	+
IAA production	−	−	−	−
HCN production	+	+	+	+
Phosphate solubilization	+	+	−	−
Ammonia production	−	−	−	+
Siderophore production	−	+	+	−

*+* present, *−* absent, *MIC* minimum inhibitory concentration of mercury, *IAA* indole acetic acid, *HCN* hydrogen cyanide.

### Growth study

**Fig. 4:**
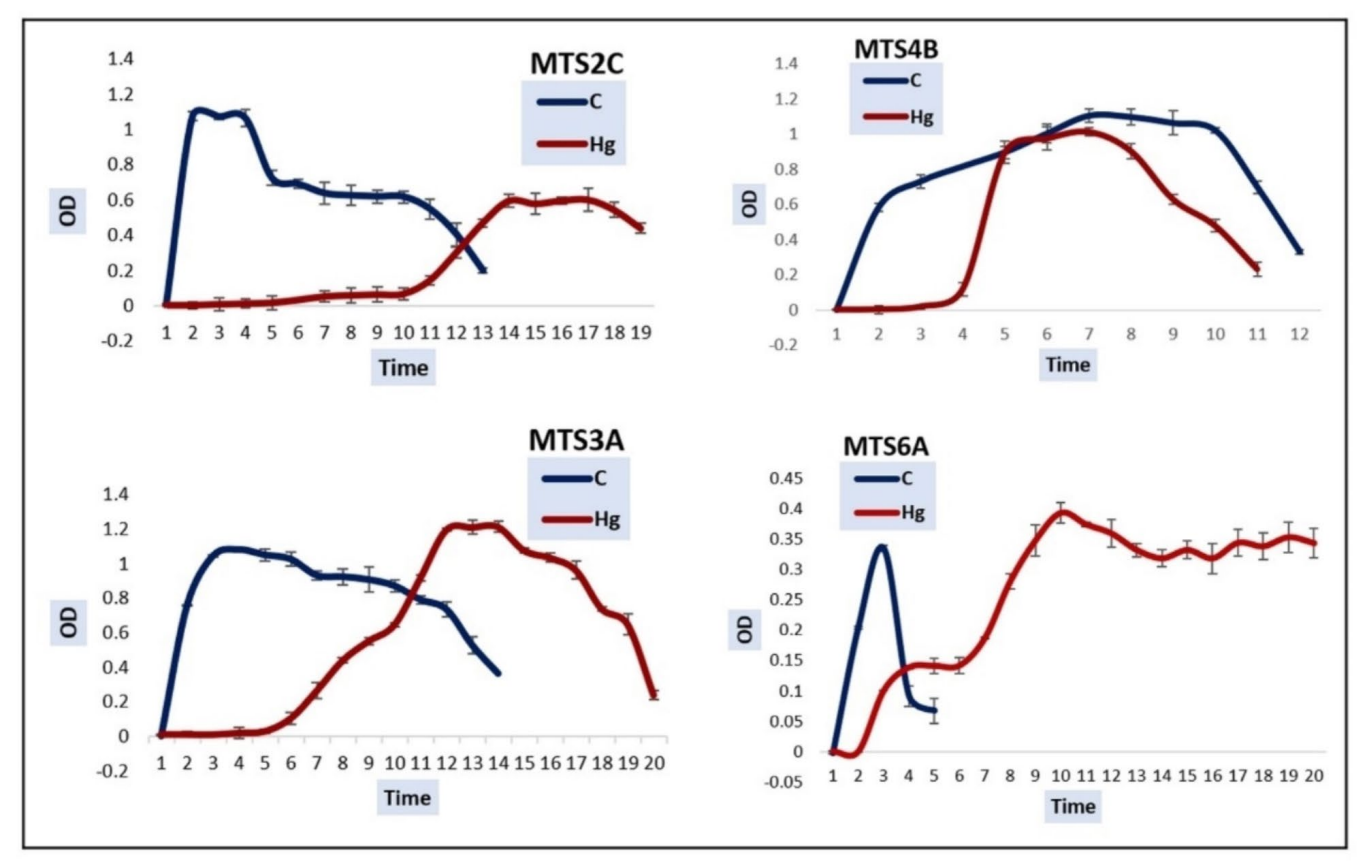
Comparative growth dynamics of selected mercury tolerant strains MTS2C, MTS3A, MTS4B, and MTS6A. The growth curve for the growth of each strain under normal conditions without the stress of mercury is represented by the data series “C”, while, the growth curve for the strain’s growth under mercury stress is represented by the data series “Hg”. The concentration of added mercury used for exerting stress for MTS2C was 0.15 mg/ml, for MTS3A was 0.1 mg/ml, for MTS4B was 0.045 mg/ml, and for MTS6A was 0.05 mg/ml, respectively. The X axis represents time lapsed in days and the Y axis represents the optical density (OD) measured over time.

The comparative growth study conducted on the selected isolates elucidated their ability to grow well under the stress of mercury (Fig. 4). While MTS2C had displayed the highest tolerance and removal capacity, the strain’s growth pattern was found to be subdued in its presence. Typical signs of stress were visible, like delayed initiation of growth, gradualization of log phase, and decreased achievable cell density. The same trends were observed with the growth pattern of MTS4B. Additionally, the entire duration of growth of MTS4B was shorter under mercury stress. The graphs plotted for MTS3A and MTS6A, on the other hand, displayed superior coping ability to the stress. These strains were able to reach a slightly higher optical density while under stress in comparison to growth under normal conditions. The results were consistent with repeated evaluation. This may be an attestation to their specialization in dealing with mercury constantly present in their environment.

### Plant growth promoting (PGP) activities

PGP traits displaying bacteria are highly desired for any bacterial species that may be introduced into a terrestrial environment^33–35^. The added capacity of plant growth promotion, beyond mercury removal, can help not only remediate the soil but also reclaim it. Such characters in these mercury tolerant strains would also be valued. The results of the assessment are displayed in Table 3. While none of the isolates were able to produce any indole acetic acid, all of them produced HCN. MTS2C and MTS3A were also phosphate solubilizing themselves. Ammonia production was only displayed by MTS6A, and siderophore production was observed in MTS3A and MTS4B. Thus, MTS3A displayed the greatest number of beneficial PGP characteristics.

### Heavy metal cross-tolerance

Polluted environments often contain more than one heavy metal contaminant. As such, it is important for remediating strains to be able to tolerate a variety of such heavy metals in order to survive and remediate diverse environments. The heavy metal cross tolerance assay was conducted to evaluate this ability in the selected isolates. According to the results in Table 4, MTS2C was able to tolerate all the heavy metals used, at both concentrations used, except cadmium and silver. In fact, silver was inhibitory at both concentrations against all the isolates. Cadmium additionally inhibited MTS6A at both concentrations and MTS4B at the higher concentration of 5 mg/ml. MTS3A shared a similar profile with MTS2C, only differing in its tolerance to chromium at 5 mg/ml. MTS4B was tolerant to lead, tin, copper, zinc, arsenic, cadmium, and nickel at 2.5 mg/ml. This tolerance only extended to lead, tin, zinc, and nickel at the increased concentration of 5 mg/ml. MTS6A displayed tolerance to the least number of heavy metals at 5 mg/ml (lead and copper) but was able to tolerate lead, tin, copper, arsenic, zinc, iron, nickel, as well as chromium at 2.5 mg/ml.

**Table 4:** Heavy metal cross-tolerance assay.

Heavy metal salt	MTS2C		MTS3A		MTS4B		MTS6A	
Concentration (mg/ml)	2.5	5	2.5	5	2.5	5	2.5	5
PbNO_3_	+	+	+	+	+	+	+	+
SnCl_2_	+	+	+	+	+	+	+	−
CuCl_2_	+	+	+	+	+	−	+	+
As_2_O_3_	+	+	+	+	+	−	+	−
ZnCl_2_	+	+	+	+	+	+	+	−
CdCl_2_	−	−	−	−	+	−	−	−
FeCl_2_	+	+	+	+	−	−	+	−
AgNO_3_	−	−	−	−	−	−	−	−
CoCl_2_	+	+	+	+	−	−	−	−
NiCl_2_	+	+	+	+	+	+	+	−
CrCl_2_	+	+	+	−	−	−	+	−

“+” = tolerant, i.e., inhibition absent; ”−” =s usceptible, i.e., inhibition present.

### Biochemical characterization and molecular identification

MTS2A, MTS3A, and MTS4B were identified as Coagulase-negative Staphylococcal Species (CoNS), viz. *Staphylococcus sciuri*,* Staphylococcus arlettae*, and *Staphyloccoccus cohnii* (Table 3). MTS6A was identified as a strain of *Lactobacillus salivarius* (*Ligilactobacillus salivarius*). The biochemical profile obtained for the isolates matched the characters described for these species in published accounts. All four isolates were catalase positive and coagulase negative, consistent with their species identity. MTS2C and MTS3A were able to perform gelatin hydrolysis, while MTS4B and MTS6A displayed starch hydrolysis activity. Only MTS2C was positive for nitrate reduction, widely associated with *Staphylococcus sciuri*, and MTS4B for urease activity. The *Staphylococcus cohnii ssp. urealyticus* typically demonstrates the ability to produce the urease enzyme, which is utilized extensively in industrial settings.

Neither *Staphylococcus sciuri*, nor *Staphyloccoccus cohnii*, have any prior reports of mercury remediation. A strain of *Staphylococcus arlettae* (MTD10A), isolated from tea garden soil of Darjeeling, has been reported to have high tolerance for mercury at 0.02 mg/ml^36^. The same tolerance was displayed by the strain MTS3A. Environmental CoNS organisms are frequently studied for their clinical relevance and antibiotic resistance due to their association with the prevalent pathogenic coagulase-positive species of *Staphylococcus aureus*^37^. However, they are largely regarded as innocuous commensal species. Tolerance to heavy metals in *Staphylococci* is only studied in association with antibiotic resistance. The operon known to be responsible for mercury tolerance (*mer* operon) has been detected on the unique *SCCmec* element in several *Staphylococcal* species^38^. *S. sciuri* is regarded as an ancestral staphylococcal species and commonly isolated from the environment, humans, and the skin and mucous membrane of animals^39^. *Lactobacillus salivarius* is generally isolated from the mouth and gastrointestinal tract of humans and animals^40^. It has been studied widely as a probiotic^41^, and its therapeutic ability to subdue the growth of pathogenic bacteria in the gastrointestinal tract^42–45^. As per our knowledge, no reports have been published on mercury tolerance exhibited by strains of *Lactobacillus salivarius*. Although, the induction of mercury tolerance from mercurial dental fillings has been studied in *Lactobacilli*^46^. The demonstration of the high mercury tolerance by the strains in this study warrant more comprehensive research into the capabilities of these environmental species.

### Antibiotic susceptibility assay

Data on the antibiotic susceptibility of environmental CoNS species is thoroughly lacking. The three CoNS species in this study, i.e., MTS2C, MTS3A and MTS4B, displayed similar antibiotic resistance against the 39 antibiotics tested (Table 5). All three were resistant to methicillin, cefoxitin, cefuroxime, ceftazidime, azithromycin, and fosfomycin. Novel determinants of fosfomycin resistance have been reported in *S. arlettae* (*fosD*), and *S. cohnii* (*fosB*)^47^. Methicillin resistance is widespread in the genus *Staphylococcus sp.*, especially well studied in *S. aureus*. However, information on the resistance patterns to β lactams is thoroughly lacking for several CoNS species including *S. arlettae*. MTS2C and MTS3A displayed resistance to both penicillin and cefoxitin, in addition to methicillin. This is an indication on the possible involvement of β-lactamase activity conferred by the *blaZ* gene commonly found in *Staphylococcus sp*. In fact, a novel β lactamase gene has been characterized in a strain of *S. arlettae* (SAN1670) called *bla*_*ARL*_^48^. CoNS species that demonstrate resistance to cefoxitin are also thought to be resistant to oxacillin and possibly carry the *mecA* gene. This can be verified by testing the isolate for reduced susceptibility to vancomycin and inducible clindamycin resistance. Such a pattern was not observed in either of the three isolates, reducing the probability of *mecA* mediated resistance. A *mecA* homologous gene has been studied in *S. sciuri*, designated *pbpD*, that provides penicillin resistance as witnessed in MTS2C. Furthermore, *mecC* gene has also been reported in *S. sciuri*.

**Table 5:** Antibiotic susceptibility assay.

Antibiotic	Exposure dose (mcg)	MTS2C	MTS3A	MTS4B	MTS6A
Cephalothin	30	S	S	S	S
Penicillin	6	R	R	S	S
Ampicillin	10	S	R	R	S
Cefoxitin	30	R	R	R	S
Cefuroxime	30	R	R	R	S
Methicillin	5	R	R	R	R
Amoxicillin	30	S	S	S	S
Ceftazidime	30	R	R	R	R
Imipenem	10	S	S	S	S
Cotrimoxazole	25	S	S	S	S
Trimethoprim	5	R	R	S	S
Ofloxacin	5	I	I	I	S
Ciprofloxacin	5	I	I	I	S
Enoxacin	10	S	S	S	S
Moxifloxacin	5	R	S	S	S
Gemifloxacin	5	R	S	S	S
Novobiocin	5	S	R	R	S
Novobiocin	30	S	I	I	S
Rifampin	5	S	R	S	S
Gentamicin	10	S	S	S	S
Streptomycin	10	S	S	S	S
Kanamycin	30	S	S	S	S
Tigecycline	15	S	S	S	S
Erythromycin	15	S	R	R	S
Azithromycin	15	R	R	R	I
Chloramphenicol	30	R	S	I	S
Fosfomycin	200	R	R	R	R
Vancomycin	30	S	I	S	S
Teicoplanin	30	S	S	S	S
Clindamycin	2	I	R	S	S
Tetracycline	30	S	R	S	S
Linezolid	30	S	S	S	S
Nitrofurantoin	300	S	S	S	S
Fusidic Acid	10	S	R	R	S
Spectinomycin	100	S	S	S	S
Polymixin B	300	S	S	S	I
Colistin	10	S	S	S	S
Pristinamycin	15	S	R	R	S
Bacitracin	8	S	S	S	I

*R* resistant, *I* intermediate, *S* susceptible.

Although, resistance to novobiocin is a characteristic feature of CoNS species, resistance was only displayed by MTS3A and MTS4B at 5 mcg. When the concentration was increased to 30 mcg, the susceptibility became inducible. Fusidic acid resistance was also only observed in these two isolates. Novel determinants for fusidic acid resistance have been described (*fusF* and *dfrC*) in *S. cohnii*. These two strains additionally displayed resistance for erythromycin and pristinamycin. Resistance to erythromycin is typically regulated by the ermC gene, one of the most widespread among *Staphylococci*. The *msrA* gene is also associated with erythromycin resistance in several *S. cohnii* strains. On the other hand, chloramphenicol resistance was only displayed by MTS2C and may be mediated by the *fexA* gene, encoding a chloramphenicol/florfenicol efflux MFS transporter. MTS2C also displayed resistance to fluroquinolones moxifloxacin and gemifloxacin, both fourth generation fluoroquinolones. The strain was instead susceptible or had inducible susceptibility to older generation fluoroquinolones. This may be due to exposure induced resistance. One of the mechanisms of resistance may be via the employment of multidrug-resistant pumps or MDRs which not only provide resistance to a host of antibiotics but also heavy metals^49^. MTS3A demonstrated resistance to tetracycline, indicating the presence of *tetL* and/or *tetK*. Resistance to rifampicin in MTS3A and trimethoprim in both MTS2C and MTS3A was also observed.

MTS6A displayed resistance to the least number of antibiotics in the study. The strain was only resistant to methicillin, ceftazidime, and fosfomycin. It also had inducible susceptibility to polymyxin B, bacitracin and azithromycin. *Lactobacillus salivarius* is widely considered a probiotic species and one of the specialized characters of probiotic strains is their ability to resist a variety of antibiotics. In recent years, distribution of transferable antibiotic resistance conferring genes among bacterial strains utilized in the food industry, has begun to be considered a hazard. This is due to the potential for such strains to transfer the antibiotic resistance property to the gut microflora of consumers. Thus, attempts are underway to utilize strains that display less antibiotic resistance, such as MTS6A. The lack of resistance in this strain may be due to its original habitat being devoid of enough exposure to antibiotics. This particular strain was isolated from the soil sample collected from the very top of Sandakphu, which may have influenced its susceptibility profile. Environmental pressures are known to play a huge role in the selection and, more so, maintenance of antibiotic resistance genes. *Lactobacillus* strains are known to be sensitive to β lactam antibiotics but demonstrate resistance to cephalosporins^50^. MTS6A, on the contrary, displayed resistance to methicillin and ceftazidime. *Lactobacilli* are also reported to be resistant to gentamicin, penicillin G, and ciprofloxacin^51^, none of which was observed for MTS6A. Information available on the antibiotic resistance in *L. salivarius* and their behavior in the environment outside of the gastrointestinal tract is rudimentary at best and requires further studies.

Antibiotic resistance mechanisms and heavy metal resistance mechanisms are often thought to be linked and co-selected on plasmids. The heavy metal resistance pattern of these isolates was far more expansive than their resistance to antibiotics, indicating more exposure to the former, than latter. Exposure to heavy metals in the environment and resultant induced resistance may in turn be responsible for the co-selection of antibiotic resistance genes in such environments. It is important to note that these strains were all isolated from an environment that receives little to no exposure to such synthetic drugs. The consistent presence of methicillin resistance, for example, among these isolates is a matter of concern. This highlights the need for more studies on environmental strains to fully understand the distribution and transfer dynamics of such resistance genes.

## Conclusion

This study provides critical evidence of mercury contamination in the high-altitude ecosystems of the Singalila National Park, particularly at Sandakphu and other elevated points along the Singalila ridge of the Eastern Himalayas. The findings show accumulation of mercury in the soil, water bodies, vegetation, and microbial communities, likely due to long-range atmospheric transport and deposition. The detection of mercury in freshly precipitated snow and sleet further supports the role of cold trapping in this region, where montane climates promote the retention of pollutants. The microbial consortia isolated from the contaminated soils demonstrated notable mercury tolerance, pointing to the adaptive responses of the local microbiota to heavy metal stress. Moreover, the identification of mercury-remediating bacterial strains with high tolerance and removal capacities offers potential for future bioremediation strategies in mercury-polluted environments. Given the ecological significance of the study area as a biodiversity hotspot, these findings raise concerns about the impact of mercury contamination on local flora, fauna, and water resources. This study emphasizes the need for further in-depth monitoring and assessment of mercury pollution in the region to fully understand the environmental and health risks posed by mercury in this vulnerable montane ecosystem.

## Methods

### Soil sampling and physicochemical characterization

Topsoil samples were collected from the highest point of Sandakphu (27°06ʹ18ʹʹN, 88°00ʹ06ʹʹE), and locations within the Singalila National Park. Seven sites were targeted with altitudes ranging between 2575 and 3636 m, as listed in Fig. 1. Each sample was a composite of five smaller pre-samples from four different corners and the diagonal bisector of an imaginary square plot with sides measuring 10 m and up to a depth of 6 cm. Collections were made in PPE (polyphenylene ether) tubes and transported to the laboratory in ice-boxes. Each sample was shade-dried in open air, crushed, homogenized and sieved through a 0.2 mm mesh. They were stored at 4 °C for further characterization. Parameters like soil pH, organic carbon (OC), organic matter (OM), sulphur content, etc. were recorded^52^. All tests were conducted in triplicate sets and the mean result obtained was considered as the final metric for each parameter for that site. A correlation analysis was performed using the mean values of each parameter against the mercury content in the soil, calculated using the correlation tool in the data analysis Toolpak in Microsoft Excel.

### Vegetation sampling

The range of elevation covered by the Singalila National Park allows for diverse vegetation types, from subtropical to subalpine. Its forests are largely known for rhododendrons, bamboo, oak, magnolia, numerous types of orchids and other wildflowers. For the purposes of this study, samples were only collected from the many different species of rhododendron (*Rhododendron sp*.) as they are widespread and have broad leaves that may accumulate more mercury from the surroundings. Samples included leaves, leaf litter, and roots, from each of the locations. One of the sites, Dhobitar, was covered in a bamboo forest. Hence, vegetation samples from this location were of bamboo (*Yushania maling*) instead of rhododendron. The samples from the highest point, i.e., Sandakphu, also included narrow leaves from silver fir trees (*Abies alba*), and ground moss vegetation samples. All collected samples were shade-dried in open air, crushed into a fine powder and homogenized before mercury quantification through ICP-MS.

### Other samples

Some water samples were collected from two prominent, high-altitude, freshwater lakes in the park, namely Tonglu and Kalipokhri. The collections were made from the surface in one fell swoop, by submerging a fresh, polypropylene tube, just under the surface of the water^53^. Sediment was also collected from the lakes. Five pre-samples of sediment were collected from different points along the littoral zone of the lakes, which were mixed to make the final sample. These were transported to the laboratory in airtight zip-loc bags placed in an ice box. At the laboratory, the samples were shade-dried in open air, homogenized and sieved through a 0.2 mm mesh before further analysis. Physicochemical characterization of these soil samples was conducted in triplicate sets. Water was also collected from a small man-made pond at Sandakphu, attached to a place of worship frequented by the very small local community. All the collections from upper Sandakphu were made on an early morning of February, after a round of snowfall had occurred overnight. This allowed for collection of freshly precipitating snow in polypropylene tubes. Days old, undisturbed precipitates of snow, referred to as “old snow”, and sleet were also collected beforehand for comparison. A final water collection was made from an undisturbed pond from the lowest altitude explored in the study, i.e., Dhotrey.

### Total mercury quantification

Mercury levels (THg) in the soil, vegetation and water samples were determined through ICP-MS^54,55^. For this process, 0.5 g (soil/vegetation) or 0.5 mL (water) of samples were measured. These underwent an initial pre-digestion with 5 mL of ultra-pure HNO_3_ (65–70%) and 1 mL of H_2_O_2_ (30%) under a fume hood for 20 min. Following this step, microwave digestion was conducted using the MDS (MARS 6, CEM, USA). The digestion protocol included an initial temperature setting of 160 °C with a 20-min ramp time and a 5-min hold, applying 600–900 W of power. The temperature was then increased to 200 °C, with a 15-min ramp and a 10-min hold at 1000–1200 W. After digestion, the MDS vessels (made of polytetrafluoroethylene) were cooled first to 50 °C and then to room temperature. The digested samples were then mixed with 0.25 mL of ultra-pure HCl (35%) and diluted to 50 mL using type I deionized water (Milli-Q, Millipore, Bedford, MA, with a resistivity of 18.2 MΩ.cm at 25 °C and a total organic carbon value below 5 ppb). These solutions were refrigerated, centrifuged at 10,000 rpm for 10 min at 20 °C, and the supernatant was analyzed using ICP-MS to measure mercury content. Each sample was prepared in triplicate. For quality assurance, NIST traceable certified reference standards (CRMs) and Standard Reference Material (SRM) 3254 green tea (QC sample) were procured.

Mercury quantification was performed using the 7800 ICP-MS (Agilent Technologies, USA), operated with Mass Hunter 5.1 software (Agilent Technologies, USA) for data acquisition and analysis. The instrument was equipped with a micromist nebulizer, quartz spray chamber, 2.5-mm injector quartz torch, off-axis ion lens assembly, and nickel cones. It utilized an octopole reaction system (ORS4), optimized to eliminate common polyatomic interferences via helium (He) collision mode and kinetic energy discrimination (KED). Instrument tuning involved using an Agilent tuning solution (P/N: 5185–5959) at low (Co^59^), mid (Y^89^), and high (Tl^205^) masses, as well as, oxide ratio (CeO/Ce) and double charge ratio (Ce^2+^/Ce) in He mode. The optimal operating conditions included: RF power at 1550 W, a sampling depth of 9.0 mm, nebulizer gas flow of 0.92 L/min, spray chamber temperature of 2.0 °C, lens tune set to autotune, He flow rate at 4.3 mL/min, KED at 3.0 V, dilution gas flow at 0.10 L/min, peak pattern with three points, 100 sweeps per replication, and a mass resolution of 1 amu. Internal standards used were scandium (Sc^45^), germanium (Ge^74^), rhodium (Rh^103^), and bismuth (Bi^209^), 200ng/mL/sample. The data was processed using MPP version 15.1 (Agilent Technologies, USA), IBM SPSS Statistics 17 (SPSS Inc., Chicago, IL, USA), and SIMCA17 (Umetrics, Sartorius, Sweden). To ensure the accuracy of the results, blanks, calibration solution, QC sample, and spiked recovery samples were analyzed regularly, with quality control checks performed after every 20 samples. The response was within ± 10% of the previous calibration. Calibration standards were prepared by serial dilution in 5% HNO_3_. Recovery percentage for mercury was 92.86% (Tested value, 0.013 mg/Kg; assigned certified value, 0.014 ± 0.001 mg/Kg). The limits of detection (LOD) and quantitation (LOQ) were calculated using the US FDA EAM 3.2 protocol.

### Mercury tolerance assessment by microbial consortia

The microbial consortia were isolated from all the collected composite topsoil samples except the two lake sediment samples. From each, 5 mg of soil was added to sterile water and vortexed to make a solution. Nutrient broth was then inoculated with 100 µl of the solution and incubated for at 30 °C for 24–48 h and observed for growth. Subsequent subcultures in nutrient broth provided the final consortium from each location. A tolerance assay was conducted to detect any tolerance that may be displayed by the consortia against mercury. For this, the 10 µl inoculum from each consortium was cultured on nutrient agar plates spiked with 0.01–0.05 mg/ml HgCl_2_. This range was based on previous studies conducted on microbial mercury tolerance capacity conducted in parts of Darjeeling^36,52^. The plates were incubated at 30 °C and observed for visible growth that would indicate a tolerance to the mercury.

### Ecological risk assessment

For the assessment of potential ecological risk based on mercury estimates found in the topsoil samples, enrichment factor (EF), geoaccumulation index (I_geo_), contamination factor (C_f_), and ecological risk factor (E_r_) were calculated. The enrichment factor was calculated to determine the effect of human activity on the concentration of mercury in the samples against a reference sample that is unpolluted. The following equation was used:

1 $$EF={\left( {Hg/Fe} \right)_{sample}}/{\left( {Hg/Fe} \right)_{crust}}$$

Where, EF = enrichment factor, (Hg/Fe)_sample_ = the ratio of Hg and reference element Fe in the sample being evaluated, (Hg/Fe)_crust_=the ratio of average Hg and Fe in the Earth’s crust. The Fe reference values used were based on previous studies on the Himalayas^56,57^. The values obtained were categorised into no enrichment (EF = < 1), minor enrichment (EF < 3), moderate enrichment (EF = 3–5), moderately high enrichment (EF = 5–10), high enrichment (EF = 10–25), very high enrichment (EF = 25–50) and extremely high enrichment (EF = > 50)^58^.

The geoaccumulation index (I_geo_) is a comparison between current concentration and preindustrial concentration of the heavy metal to evaluate the level of pollution present at a location. It was calculated using the following equation:

2 $${I_{geo}}=Lo{g_2}[{C_i}/(1.5x{C_{ri}})]$$

Where, I_geo_=geoaccumulation index, C_i_=concentration of mercury in sample (mg/kg), and C_ri_=the element’s concentration in shale^59^. The geoaccumulation index is usually classified into six distinct categories that were used for the analysis of obtained data, namely, unpolluted (I_geo_<0), moderately polluted (0 ≤ I_geo_<1), moderately to heavily polluted (1 ≤ I_geo_<2), heavily polluted (2 ≤ I_geo_<3), heavily to extremely polluted (3 ≤ I_geo_<4), and very highly polluted (4 ≤ I_geo_<5)^60^.

Contamination factor was used to determine the degree of contamination in the soil samples collected. The following equation was used for calculation:

3 $${C_f}=C{m_{sample}}/C{m_{shale}}$$

Where, C_f_=contamination factor, Cm_sample_=Hg concentration in the soil sample being evaluated, and Cm_shale_= the element’s concentration in shale^59^. The results obtained were categorized into low contamination (C_f_ < 1), moderate contamination (1 ≤C_f_ < 3), considerable contamination (3 ≤ C_f_ < 6), and very high contamination (C_f_ ≥ 6)^61^.

The ecological risk factor is used to determine the potential for harm to plants and animals in an environment contaminated with heavy metals. In this case, it was calculated using the following equation:

4 $${E_r}={T_r} \times {\text{ }}{C_f}$$

Where, E_r_=ecological risk factor of the sample being evaluated, T_r_=toxicity response factor of Hg (= 40)^59^, and C_f_=contamination factor previously calculated for each sample. The defined limits for potential ecological risk factor for heavy metals is categorised as low risk (E_r_<40), moderate risk (E_r_=40–80), moderate to the high risk (E_r_=80–160), high risk (E_r_=160–320), and very-high risk (E_r_>320)^61^.

### Isolation of mercury-remediating bacterial strains

Pure culture isolation was conducted for the visible bacterial colonies exhibiting tolerance to mercury within each consortium via repeated streaking on nutrient agar plates supplemented with HgCl_2_ (0.01 mg/ml). The concentration of mercury used is based on internal standards used in our laboratory for the isolation of “high tolerance” bacterial strains from Darjeeling, determined from extensive studies on the mercury tolerant bacterial population of the region. The disc diffusion method was employed using HgCl_2_ soaked discs of varying concentrations (0.01–1 mg/ml) to determine the minimum inhibitory concentration (MIC) of mercury against the final isolates. The mercury removal capacity of select isolates was also estimated after incubation in a mercury containing medium. A known concentration of 0.01 mg/ml (10 ppm) of HgCl_2_ was added to the nutrient broth and inoculated with the isolates. Following incubation for 48 h at 30 °C, the broth was centrifuged at 7000 rpm for 10 min and the supernatant was collected. Residual mercury of the supernatant was determined through ICP-MS, and the difference between initial and final metal concentration in the media elucidated the mercury removal capacity of the isolates under nutrient rich conditions.

### Growth study

The growth pattern of the isolates under mercury stress was monitored in nutrient broth supplemented with HgCl_2_ at a concentration of half their respective MICs as listed in Table 3. The broths were inoculated with overnight cultures of the isolates and incubated at 35 °C. The optical density (OD) was noted after regular 24 h intervals at 600 nm using spectrophotometer (Agilent Cary 60 UV–Vis). Broth devoid of mercury was used for comparison. The data obtained was finally plotted in graphical form to reveal the comparative growth pattern of the isolates.

### Plant growth promoting (PGP) activities

Qualitative tests were carried out for the production of indole acetic acid (IAA)^62^, hydrogen cyanide (HCN) and ammonia^63^. Their phosphate solubilizing ability evaluated by streaking on Pikovskayas agar and observing for production of a halo. The detection of siderophores was done following the SD-CASA plate assay^64^.

### Heavy metal cross-tolerance

Tolerance towards other heavy metals was studied via disk diffusion method using metal salts at concentrations of 2.5 and 5 mg/ml^36,52,65^. Filter paper discs were submerged into the metal salt solutions and placed onto nutrient agar plates inoculated with the bacterial culture using spread plate technique. The metals used were lead, tin, arsenic, copper, cadmium, iron, zinc, cobalt, nickel, chromium and silver. The corresponding nitrate or chloride or oxide salts were used. Susceptibility was determined by recording inhibition zones for each metal. The test was conducted to evaluate bacterial efficacy in the face of other stress factors that may be present in a contaminated environment.

### Biochemical characterization and molecular identification

Several standard biochemical tests were performed (as listed in Table 3). Molecular identification was only performed for three of the strains displaying the highest tolerance as well as robust growth pattern in the presence of mercury. Genomic DNA was isolated from these isolates. Quality was evaluated on 1.8% Agarose Gel. Isolated DNA was amplified with 16 S rRNA Specific Primer (27 F and 1492R) using a Veriti^®^ 96 well Thermal Cycler to obtain a single discrete PCR amplicon band of ~ 1500 bp. The PCR amplicon was bead purified and further subjected to Sanger sequencing. The bi-directional DNA sequencing reaction of the PCR amplicon was carried out with 27 F and 1492R primers using the BDT v3.1 Cycle sequencing kit on the ABI 3500Dx Genetic Analyzer. GeneTool was used to generate a consensus sequence of 16 S rRNA. BLAST analysis was done against sequences available in the NCBI GenBank database.

### Antibiotic susceptibility assay

The isolates were tested for susceptibility to commonly used, synthetic, semi-synthetic, and natural antibiotics with antibiotic discs purchased from HiMedia, India. The test was conducted via the single disc diffusion method on Mueller Hinton agar for clear visualization. Assessment of inhibition zones was as per interpretive criteria provided by the antibiotic disc manufacturer (HiMedia), CLSI (2020)^66^ as well as available data on cases of resistance in published literature. Accordingly, they were classified as Susceptible (S), Intermediate (I) and Resistant (R). Heavy metal resistance displayed by clinical bacterial strains have often been seen to display antibiotic resistance as well, due to co-selection of genes. The antibiotic susceptibility assay was conducted to see whether high tolerance to mercury was accompanied by high incidence of antibiotic resistance among the strains as well.

## Electronic supplementary material

Below is the link to the electronic supplementary material.

Supplementary Material 1

## Data Availability

The datasets generated and/or analysed during the current study are available in the GenBank repository, under the accession numbers PP415850, PP436698, PP416278, and PP815117.
