# Supplementary material for: Uncovering mercury accumulation and the potential for bacterial bioremediation in response to contamination in the Singalila National Park
Source: Sci Rep. 2025 Jan 29;15:3664. doi: 10.1038/s41598-024-81927-5 (PMC11779926; doi:10.1038/s41598-024-81927-5)
Supplement: Supplementary file 1 — Supplementary Material 1 [file 41598_2024_81927_MOESM1_ESM.docx]

| Location | Type | Sample |
| --- | --- | --- |
| Upper Sandakphu | Soil | Topsoil (0-10 cm) |
|  | Vegetation | Broad leaves *(Rhododendron* *sp*.) |
|  |  | Narrow leaves (*Abies* *alba*) |
|  |  | Roots |
|  |  | Litterfall |
|  |  | Moss |
|  | Waterbody | Manmade pond water |
|  | Precipitation | Freshly precipitating snow |
|  |  | Older snow deposit (2-4 days old) |
|  |  | Sleet |
| Lower Sandakphu | Soil | Topsoil (0-10 cm) |
|  | Vegetation | Leaf (*Rhododendron* *sp*.) |
|  |  | Roots |
|  |  | Litterfall |
| Kalipokhri | Soil | Topsoil (0-10 cm) |
|  | Vegetation | Leaf (*Rhododendron* *sp*.) |
|  |  | Roots |
|  |  | Litterfall |
|  | Waterbody | Lake surface water |
|  |  | Lake sediment (littoral zone) |
| Dhopitar | Soil | Topsoil (0-10 cm) |
|  | Vegetation | Leaf (*Yushania* *maling*) |
|  |  | Roots |
|  |  | Litterfall |
| Tonglu | Soil | Topsoil (0-10 cm) |
|  | Vegetation | Leaf (*Rhododendron* *sp*.) |
|  |  | Roots |
|  |  | Litterfall |
|  | Waterbody | Lake surface water |
|  |  | Lake sediment (littoral zone) |
| Gairibas | Soil | Topsoil (0-10 cm) |
|  | Vegetation | Leaf (*Rhododendron* *sp*.) |
|  |  | Roots |
|  |  | Litterfall |
| Dhotrey | Soil | Topsoil (0-10 cm) |
|  | Vegetation | Leaf (*Rhododendron* *sp*.) |
|  |  | Roots |
|  |  | Litterfall |
|  | Waterbody | Pond surface water |
